# The Influence of Environmental Factors on the Prevalence of Myopia in Poland

**DOI:** 10.1155/2017/5983406

**Published:** 2017-11-19

**Authors:** Maciej Czepita, Damian Czepita, Wojciech Lubiński

**Affiliations:** 2nd Department of Ophthalmology, Pomeranian Medical University, al. Powstańców Wlkp. 72, 70-111 Szczecin, Poland

## Abstract

**Purpose:**

In the paper, we describe and discuss the results of epidemiological studies concerning myopia carried out in Poland.

**Materials and Methods:**

Results from the examination of 5601 Polish school children and students (2688 boys and 2913 girls) aged 6 to 18 years were analyzed. The mean age was 11.9 ± 3.2 years. Every examined student had undergone the following examinations: distance visual acuity testing, cover test, anterior segment evaluation, and cycloplegic retinoscopy after instillation of 1% tropicamide, and a questionnaire was taken.

**Results:**

We have found that (1) intensive near work (writing, reading, and working on a computer) leads to a higher prevalence of myopia, (2) watching television does not influence the prevalence of myopia, and (3) being outdoors decreases the prevalence of myopia.

**Conclusions:**

The results of our study point to insufficiency of accommodation contributing to the pathogenesis of myopia.

## 1. Introduction

Myopia is a major and still unresolved health problem in the world. It is currently estimated that more than 22% of the world population has myopia. This means that 1.5 billion people have myopia. In East Asian countries, the prevalence of myopia is at 70–80%. In Western countries, 25–40% has myopia. In the United States, the number of myopes has double in the past 30 years [1–3].

Myopia is determined by genetic and environmental factors [4]. Environmental factors include reading, writing, and visual work when using a computer. Some researchers believe that even watching television has an influence on the development of myopia [5–17]. It is currently believed that outdoor activity leads to a lower prevalence of myopia [10, 13, 14, 18–35].

Research into the epidemiology of myopia is ongoing throughout the entire world [1–3, 5–31]. In Poland, the greatest achievements in myopia research belong to the Pomeranian Medical University in Szczecin [32, 33]. That is why we decided to present our results.

## 2. Materials and Methods

In this paper, we describe and discuss the results of epidemiological studies concerning myopia carried out in Szczecin, Poland. Special attention was put on the role of reading, writing, and visual work using a computer and outdoor activity.

The studies were carried out from October 2000 till March 2009. Results from the examination of 5601 Polish school children and students (2688 boys and 2913 girls) aged 6 to 18 years were analyzed. The mean age was 11.9 ± 3.2 years. The students examined were Caucasian, and there were no children of mixed ethnicity. Every examined student had undergone the following examinations: distance visual acuity testing, cover test, anterior segment evaluation, and cycloplegic retinoscopy after instillation of 1% tropicamide, and a questionnaire was taken. The methodology of the examination has been described in details in previous works as follows. Participation was voluntary. Before beginning the examinations, the doctors met with the children, their parents, or legal guardians and teachers. It was explained what the examinations were about. The children, parents, or legal guardians and teachers had an opportunity to discuss the study with the experimenters prior to giving consent. Informed consent as well as date of birth was obtained in each case from children, parents, or legal guardians and school principals. The studies were approved by the Bioethics Committee of the Pomeranian Medical University. The research protocol adhered to the provisions of the Declaration of Helsinki for research involving human subjects.

Every examined person underwent retinoscopy under cycloplegia. Cycloplegia was induced with two drops of 1% tropicamide administered 5 min apart. Thirty minutes after the last drop, pupil dilation and the presence of light reflex was evaluated as later retinoscopy was performed. Retinoscopy was performed in darkened school's consulting rooms.

The refractive error readings were reported as a spherical equivalent (SE) (sphere power plus half-negative cylinder power). Hyperopia was defined to be spherical equivalent higher than +0.5 D and emmetropia to be higher than −0.5 and lower than +0.5 D. Myopia was defined to be with a SE lower than −0.5 D. Astigmatism did not exceed 0.5 DC. The mean SE was calculated after examination of both eyes [32, 33].

## 3. Results

**Figure 1:**
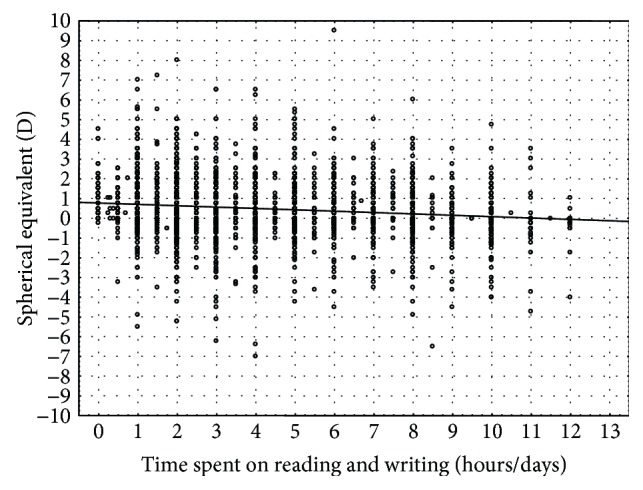
Mean spherical equivalent in relation to reading and writing.

After having examined the 5601 students, it has been shown that reading and writing lead to a higher prevalence of myopia (*p* < 0.000001) [32] (Figure 1).

**Figure 2:**
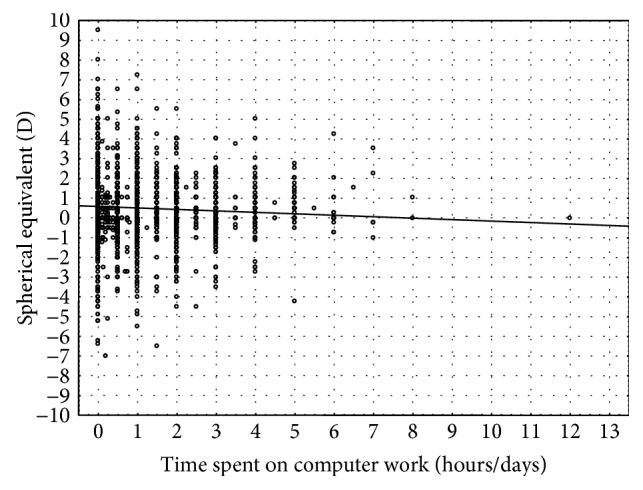
Mean spherical equivalent in relation to using a computer.

It has also been observed that working on a computer leads to a higher prevalence of myopia (*p* < 0.000001) [32] (Figure 2).

**Figure 3:**
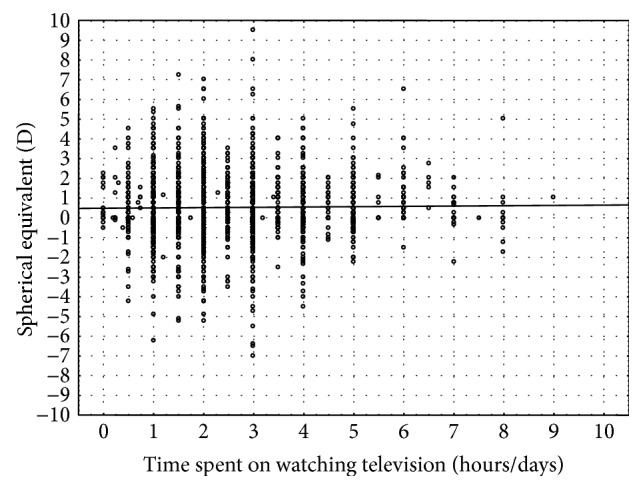
Mean spherical equivalent in relation to watching television.

It has been shown that watching television does not have an influence on the prevalence of myopia (*p* = 0.31) [32] (Figure 3).

**Figure 4:**
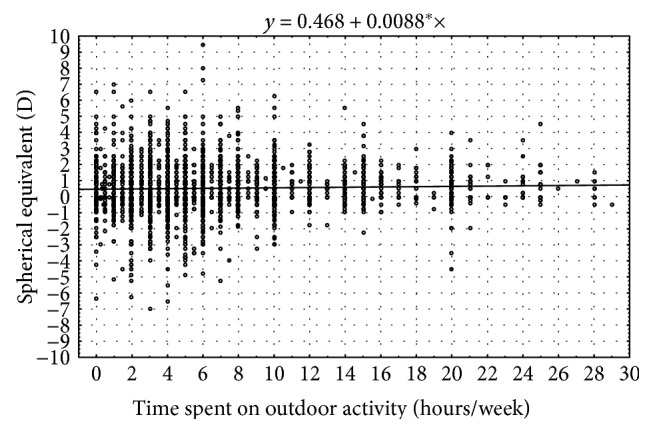
Mean spherical equivalent in relation to outdoor activity.

Outdoor activity however leads to a lower prevalence of myopia (*p* < 0.007) [33] (Figure 4).

## 4. Discussion

Opinions concerning the influence of reading, writing, and visual work when using a computer, watching television, and outdoor activity are varied. Most authors accept that reading, writing, and visual work when using a computer lead to a higher prevalence of myopia. However, some authors debate these relationships. Concerning watching television, most authors believe that it does not have an influence on the development of myopia (Table 1). Outdoor activity however decreases the prevalence of myopia (Table 2) [3, 4, 32, 33].

It is accepted that the higher prevalence of myopia due to reading, writing, and visual work using a computer are attributed to insufficiency of accommodation during visual near work. It has also been observed that spasms of accommodation are considered the factors of myopia [4]. The results of these studies were confirmed by researchers from the Pomeranian Medical University in Szczecin, Poland, by achieving a coefficient of statistical significance *p* < 0.000001 [32].

During the years of 2005-2006, Buehren et al. [34] and Collins et al. [35] have showed that reading and visual work when using a computer leads to a change in the shape of the cornea, which may lead to the development of myopia. The results obtained by the authors are in agreement with the hypothesis that lid-induced corneal aberrations may play a significant role in the development of myopia.

Most authors believe that watching television does not cause myopia. The argument behind this belief is that watching television usually from a few meters away does not cause insufficiency of accommodation [4]. Research done at the Pomeranian Medical University in Szczecin, Poland, has also proved that watching television does not lead to a higher prevalence of myopia (*p* = 0.31) [32]. However, it happens that watching television does lead to a quicker development of myopia when the television monitor is placed too close to the eye [4].

Currently, it is accepted that outdoor activity leads to a lower prevalence of myopia. This is probably due to the fact that during distant visual work, there is no spasm of accommodation [3, 4]. This relationship has been also proven by research carried out at the Pomeranian Medical University in Szczecin, Poland, achieving a coefficient of statistical significance of *p* < 0.007 [33].

It also has to be added that the results of our research are reliable because they have been conducted on a large and homogenous group of people of the Caucasian race. Besides, our research was done after cycloplegia.

## 5. Conclusions

The results of the examinations show that insufficiency of accommodation has a role in the pathogenesis of myopia.

## Figures and Tables

**Table 1:** Dependency between reading, writing, using a computer, or watching TV and myopia.

Reference	Country	Dependency between reading and writing and myopia	Dependency between using a computer and myopia	Dependency between watching TV and myopia
Cole et al. [5]	Australia		+	
Czepita et l. [6]	Poland	+	+	
Giloyan et al. [7]	Armenia	+		
Khader et al. [8]	Jordan	+	+	
Kinge et al. [9]	Norway	+		
Konstantopoulos et al. [10]	Greece	+	+	
Li et al. [11]	China	+		+
Mutti et al. [12]	U.S.A.	+		
Pärssinen et al. [13]	Finland	+		+
Saw et al. [14]	China	+		
Saxena et al. [15]	India	+	+	+
Wong et al. [16]	Hong Kong	+		
You et al. [17]	China	+	+	+

**Table 2:** Dependence between outdoor activity and myopia.

Reference	Country
Dirani et al. [18]	Singapore
French et al. [19]	Australia
Guggenheim et al. [20]	UK
Guo et al. [22]	China
Guo et al. [23]	China
Guo et al. [21]	China
Jacobsen et al. [24]	Denmark
Jones et al. [25]	U.S.A.
Lin et al. [26]	China
Mutti et al. [12]	U.S.A.
Ngo et al. [27]	Singapore
Pärssinen et al. [13]	Finland
Rose et al. [28]	Australia
Saxena et al. [15]	India
Shah et al. [29]	UK
You et al. [17]	China
Wu et al. [30]	Taiwan
Zhou et al. [31]	China
